# Pre-diagnostic biomarkers of metabolic dysregulation and cancer mortality

**DOI:** 10.18632/oncotarget.24559

**Published:** 2018-02-23

**Authors:** Tomi Akinyemiju, Justin Xavier Moore, Suzanne E. Judd, Maria Pisu, Michael Goodman, Virginia J. Howard, Leann Long, Monika Safford, Susan C. Gilchrist, Mary Cushman

**Affiliations:** ^1^ Department of Epidemiology, University of Alabama at Birmingham, Birmingham, AL, USA; ^2^ Comprehensive Cancer Center, University of Alabama at Birmingham, Birmingham, AL, USA; ^3^ Department of Epidemiology, University of Kentucky, Lexington, KY, USA; ^4^ Department of Biostatistics, University of Alabama at Birmingham, Birmingham, AL, USA; ^5^ Division of Preventive Medicine, University of Alabama at Birmingham, Birmingham, AL, USA; ^6^ Department of Epidemiology, Emory University Rollins School of Public Health, Atlanta, GA, USA; ^7^ Department of Medicine, Weill Cornell Medical College, New York, NY, USA; ^8^ Department of Clinical Cancer Prevention and Cardiology, The University of Texas MD Anderson Cancer Center, Houston, TX, USA; ^9^ Department of Medicine and Vermont Cancer Center, Larner College of Medicine at the University of Vermont, Burlington, VT, USA

**Keywords:** metabolic biomarkers, cancer mortality, racial disparities, metabolism

## Abstract

**INTRODUCTION:**

The obesogenic milieu is a pro-tumorigenic environment that promotes tumor initiation, angiogenesis and metastasis. In this prospective cohort, we examined the association between pre-diagnostic metabolic biomarkers, plasma adiponectin, resistin, leptin and lipoprotein (a), and the risk of cancer mortality.

**METHODS:**

Prospective data was obtained from the REasons for Geographic and Racial Differences in Stroke (REGARDS) cohort of Blacks and Whites followed from 2003 through 2012 for cancer mortality. We determined the association between metabolism biomarkers (log-transformed and tertiles) and risk of cancer mortality using Cox Proportional Hazards models with robust sandwich estimators to calculate the 95% confidence intervals (CIs), and adjusted for baseline covariates, including age, gender, income, education, physical activity, BMI, smoking status, alcohol use, and comorbidity score.

**RESULTS:**

Among 1764 participants with available biomarker data, each SD higher log-leptin was associated with a 54% reduced risk of total cancer mortality (HR: 0.46, 95% CI: 0.23 – 0.92) and obesity-related cancer mortality (HR: 0.55, 95% CI: 0.39-0.79). Among Blacks only, each SD higher log-resistin was associated with a nearly 7-fold increased risk of cancer mortality (adjusted HR: 6.68, 95% CI: 2.10 – 21.21). There were no significant associations of adiponectin or Lp(a) and cancer mortality.

**CONCLUSIONS:**

Leptin is involved in long-term regulation of energy balance, while resistin is involved in chronic inflammation and LDL production. These findings highlight the biological mechanisms linking metabolic dysregulation with cancer mortality, and the influence of resistin on cancer mortality only among Blacks suggests that this hormone may be a useful biomarker of racial differences in cancer mortality that deserves further study.

**IMPACT:**

Our observed increased risk of cancer mortality associated with higher serum resistin levels among Blacks suggests that if validated in larger cohorts, clinical strategies focused on resistin control may be a promising cancer prevention strategy.

## INTRODUCTION

The obesogenic milieu induces alterations in critical metabolic biomarkers such as adiponectin, resistin, leptin, and lipoprotein (a) [Lp(a)]. This environment promotes tumor initiation, angiogenesis and metastasis [1–4]. A recent study observed that patients in the highest quartile of pre-diagnostic plasma adiponectin had a nearly 90% higher risk of colorectal cancer mortality compared with those in the lowest quartile [1]. Other studies have also reported an association between higher leptin and resistin levels at diagnosis and increased cancer aggressiveness- defined as disease spread to sentinel lymph nodes [3]- and higher stage at diagnosis [5]. Racial disparities in mortality rates for most cancer types are well documented, placing Black patients at higher risk of adverse outcomes compared to Whites in the U.S, a trend that has remained consistent over the past several decades. While the cause of the disparity remains poorly understood, it is most likely a complex interplay of factors relating to socioeconomics, access to healthcare, biological factors including burden of comorbidities and genetic factors. Examination of racial differences in cancer-related biomarkers may provide useful clues to understanding biological mechanisms leading to higher cancer mortality among Blacks.

While the documented risk factors for cancer mortality include obesity, smoking and alcohol use [6–9], metabolic dysregulation is hypothesized to play a significant role in the etiology, metastasis rate and mortality due to cancer [10]. Racial disparities exist in the prevalence of these risk factors, especially obesity, [10]. Nevertheless, it remains unclear whether increased adiposity is an independent risk factor for cancer mortality, or whether other metabolic consequences of obesity are most important for increased risk, and racial disparities in, cancer mortality [11]. In this analysis, we examined whether biomarkers of metabolic dysregulation, measured by pre-diagnostic plasma adiponectin, resistin, leptin and Lp(a), increased the risk of cancer mortality differentially by race in a prospective cohort of Blacks and Whites after adjusting for baseline socio-demographics and other risk factors. We conducted subgroup analysis focusing on common obesity related cancers (e.g. breast, colorectal, pancreatic, endometrial), and conducted stratified analysis examining the association between metabolic biomarkers and cancer mortality by obesity status to assess whether this association is stronger among this group of participants.

## RESULTS

The distributions of baseline adiponectin, leptin, Lp(a), and resistin measures in the study cohort are presented in Table 1. Blacks were more likely to be in the lowest tertile for adiponectin (60.4% vs. 34.3%, p <0.01) and resistin (52.0% vs. 49.4%, p<0.01), but in the highest tertile for leptin (60.6% vs. 31.6%, p<0.01) and Lp(a) (58.7% vs. 23.6%, p<0.01). Higher resistin was associated with low physical activity (41.7% vs. 28.0%, p <0.01), coronary artery disease (20.0% vs. 13.6%, p<0.01), diabetes (26.1% vs. 21.9%, p<0.01), and dyslipidemia (62.5% vs. 57.1%, p<0.01). Higher leptin was associated with lower education and income, and higher BMI (32.6 vs. 25.0, p<0.01). Participants with higher Lp(a) were more likely to report hypertension (65.0% vs. 53.5%, p<0.01) than those with lower levels (Table 2).

**Table 1:** Distribution of metabolic biomarkers by tertiles in the study cohort

	N	1^st^ Tertile	N	2^nd^ Tertile	N	3^rd^ Tertile	Median (IQR)
**Biomarker**							
Adiponectin (ng/ml)	588	0.76 – 7.83	588	7.86 – 16.65	588	16.65 – 186.35	11.46 (13.92)
Log Adiponectin	588	6.63 – 8.97	588	8.97 – 9.72	588	9.72 – 12.14	3.21 (0.56)
Resistin (pg/ml)	588	1.76-20.40	588	20.41-29.61	588	29.64-360.25	24.77 (14.17)
Log Resistin	588	0.56 – 3.02	588	3.02 – 3.39	588	3.39 – 5.89	9.35 (1.17)
Leptin (ng/ml)	588	0.25 – 10.70	588	10.70 – 28.32	588	28.39 – 224.61	17.83 (27.07)
Log Leptin	588	5.54 – 9.28	588	9.28 – 10.25	588	10.25 – 12.32	9.79 (1.50)
Lpa (mg/dl)	576	0.02 – 0.12	600	0.13 – 0.41	588	0.42 – 2.17	0.25 (0.43)
Log Lp(a)	576	−3.91, −2.12	600	−2.04, −0.89	588	−0.87, 0.77	−1.39 (−2.94)

**Table 2:** Baseline characteristics of REGARDS participants by tertiles of metabolic biomarkers

	Tertiles of Metabolic Biomarkers							
	Adiponectin (pg/ml)		Resistin (pg/ml)		Leptin (pg/ml)		Lp(a) (mg/dl)	
	T1	T3	T1	T3	T1	T3	T1	T3
**Participants**	588	588	588	588	588	588	576	588
**Weighted Participants**	8386	7946	8434	7145	7750	8578	8404	7899
	**Presented as Column % or Median (IQR)**							
**Age at baseline, Median (IQR)**	64 (57-71)	71 (62-79)	65 (57-72)	70 (61-78)	68 (59-77)	67 (59-75)	68 (59-76)	67 (59-75)
**Black Race, %**	60.4	34.3	52.0	49.4	31.6	60.6	23.6	58.7
**Male Gender, %**	61.8	29.5	47.0	41.4	79.7	12.2	48.9	62.5
**Education < High School, %**	14.5	11.8	14.6	15.1	11.0	18.1	9.3	17.6
**Income <$20,000, %**	18.2	15.4	16.6	18.4	11.2	25.2	14.6	20.1
**No Exercise Activity, %**	34.0	33.8	28.0	41.7	23.8	43.4	34.6	32.7
**BMI (kg/m^2^), Median (IQR)**	29 (27-34)	27 (24-31)	28 (25-32)	29 (25-32)	25 (23-27)	33 (29-37)	28 (25-32)	29 (25-33)
**Current Smoking Status, %**	18.2	11.6	13.4	15.2	20.4	10.8	16.0	13.2
**Heavy Alcohol Consumption, %**	0.9	5.5	4.9	1.9	6.8	1.7	3.7	3.1
**Medication Use, %**								
NSAIDs – Aspirin	48.2	39.2	45.3	44.7	43.5	44.8	44.1	46.6
Statins	38.5	34.6	31.5	36.4	29.3	42.2	31.6	40.5
**Comorbid Conditions, %**								
Atrial fibrillation	11.9	8.9	9.5	10.7	5.5	11.6	11.2	8.6
Chronic lung disease	7.0	7.1	5.6	6.5	4.6	8.3	9.1	7.4
Coronary artery disease	20.2	16.0	13.6	20.0	17.4	15.3	16.4	19.1
Deep vein thrombosis	5.1	7.7	6.3	7.4	3.7	7.9	4.8	8.0
Diabetes	25.7	22.7	21.9	26.1	14.2	29.3	24.5	25.8
Dyslipidemia	72.6	49.2	57.1	62.5	60.9	60.4	63.3	64.0
Hypertension	62.6	53.9	54.0	71.8	50.8	76.8	53.5	65.0
Myocardial infarction	14.8	13.4	11.0	14.9	14.1	10.6	12.2	14.3
Peripheral artery disease	0.8	3.2	2.3	1.6	2.6	1.0	1.2	3.0
Stroke	7.0	4.2	5.1	7.9	3.7	7.2	5.2	6.4
**Comorbidity Score, Mean (SD)**	2.33 (1.44)	2.02 (1.53)	2.01 (1.43)	2.40 (1.53)	1.89 (1.47)	2.39 (1.46)	2.10 (1.57)	2.31 (1.46)

% - Denotes column percentages.

IQR – Interquartile range.

Comorbidity score is total of comorbidities, presented as mean and standard deviation (SD).

**Figure 1:**
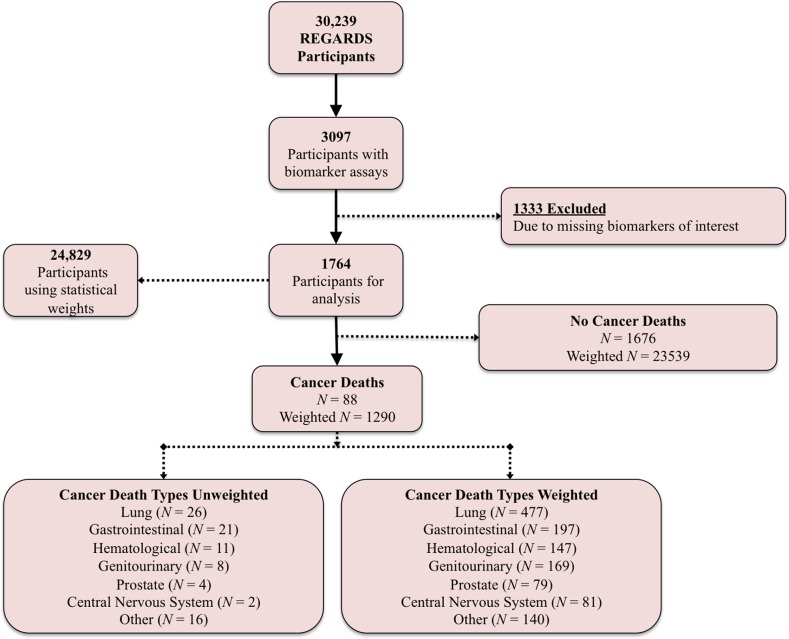
REGARDS study breakdown

A total of 1764 case-cohort sample participants representing 24,829 REGARDS participants with relevant baseline data were included in the analysis of cancer mortality, and 88 cancer deaths were observed, representing approximately 1290 (5.2%) cancer deaths among participants from 2003 through 2012 (Figure 1). The most common cancer types were lung (n = 26 (29.5%), weighted n = 477 (37.0%)), gastrointestinal (n = 21 (23.9%), weighted n = 197 (15.3%)), hematological (n = 11 (12.5%), weighted n = 147 (11.4%)), and genitourinary (n = 8 (9.1%), weighted n = 169 (13.1%)). About 30% of all cancer deaths were obesity-related cancers.

### Risk of cancer mortality

As shown in Table 3, higher leptin was associated with a reduced risk of cancer mortality in unadjusted models, an association that was maintained in the adjusted models. Higher leptin reduced the risk of cancer mortality by about 54% per SD log increase (adjusted HR per SD log leptin: 0.46, 95% CI: 0.23 – 0.92). The other metabolism related biomarkers, adiponectin, resistin and Lp(a) were not associated with cancer mortality in the entire cohort. In race stratified analysis (Table 4), among Blacks, higher resistin levels were associated with more than a 6-fold increased risk of cancer mortality with full adjustment (adjusted HR per SD log resistin: 6.68, 95% CI: 2.10-21.21). In addition, higher leptin levels in Blacks were also associated with reduced risk of cancer mortality after adjusting for confounders (adjusted HR per SD log leptin: 0.26, 95% CI: 0.13-0.51). There were no significant associations between metabolic biomarkers and cancer mortality among White participants. When focused on obesity-related cancer mortality only (Table 5), higher leptin levels were associated with reduced risk of obesity-related cancer mortality (adjusted HR per SD log leptin: 0.59, 95% CI: 0.47-0.75), an association that was modestly attenuated after adjustment for baseline health behaviors and comorbidities. There were no significant associations between adiponectin, resistin and Lp(a) and obesity-related cancer mortality.

**Table 3:** Hazard ratios (HR) and 95% confidence intervals (CI) of cancer death by baseline metabolic biomarker among all participants

	Log-Transformed	Tertile 1	Tertile 2	Tertile
**Adiponectin (pg/ml), N (Cases)**		588 (27)	588 (23)	588 (38)
**Weighted N (Cases)**		8386 (525)	8497 (381)	7946 (384)
Crude	1.21 (0.93-1.57)	Referent	0.78 (0.44-1.38)	1.48 (0.88-2.48)
Model 1	1.00 (0.66-1.53)	-	0.67 (0.27-1.70)	0.81 (0.34-1.90)
Model 2	0.91 (0.60-1.38)	-	0.63 (0.25-1.55)	0.72 (0.30-1.73)
Model 3	0.83 (0.59-1.17)	-	0.55 (0.24-1.28)	0.57 (0.28-1.18)
**Resistin (pg/ml), N (Cases)**		588 (24)	588 (30)	588 (34)
**Weighted N (Cases)**		8434 (336)	9250 (534)	7145 (420)
Crude	1.65 (0.98-2.76)	Referent	1.13 (0.65-1.98)	1.71 (0.99-2.97)
Model 1	1.30 (0.57-2.98)	-	1.18 (0.43-3.27)	1.23 (0.53-2.89)
Model 2	1.51 (0.66-3.46)	-	1.25 (0.44-3.57)	1.44 (0.60-3.45)
Model 3	2.05 (0.87-4.83)	-	1.58 (0.68-3.70)	1.79 (0.82-3.88)
**Leptin (pg/ml), N (Cases)**		588 (45)	588 (26)	588 (17)
**Weighted N (Cases)**		7750 (557)	8501 (423)	8578 (310)
Crude	**0.73 (0.61-0.87)**	Referent	**0.55 (0.33-0.91)**	**0.38 (0.21-0.68)**
Model 1	**0.41 (0.28-0.61)**	-	**0.33 (0.16-0.69)**	**0.21 (0.08-0.52)**
Model 2	**0.41 (0.26-0.64)**	-	**0.37 (0.18-0.76)**	**0.22 (0.08-0.59)**
Model 3	**0.46 (0.23-0.92)**	-	1.26 (0.42-3.79)	0.72 (0.16-3.32)
**Lp(a) (mg/dl), N (Cases)**		576 (36)	600 (25)	588 (27)
**Weighted N (Cases)**		8404 (497)	8527 (412)	7899 (381)
Crude	0.86 (0.70-1.06)	Referent	0.66 (0.39-1.13)	0.79 (0.47-1.33)
Model 1	1.01 (0.77-1.32)	-	**0.53 (0.28-0.98)**	1.37 (0.64-2.92)
Model 2	1.04 (0.75-1.43)	-	0.52 (0.25-1.06)	1.42 (0.54-3.70)
Model 3	1.28 (0.91-1.81)	-	0.90 (0.41-1.99)	1.87(0.73-4.81)

Model 1: Adjusted for age, gender, education, income, and site.

Model 2: Additionally adjusted for race.

Model 3: Additionally adjusted for exercise activity, BMI, smoking status, alcohol use, and comorbidity score.

T1: 1^st^ tertile; T2: 2^nd^ tertile; T3: 3^rd^ tertile

Bold indicates significance at 0.05 alpha level.

**Table 4:** Hazard ratios (HR) and 95% confidence intervals (CI) of cancer deaths by metabolic biomarkers, stratified by race

		Black participants (N = 830, weighted N = 11,200)			White participants (N = 934, weighted N = 13,629)	
	p-value interaction^1^	Log-Transformed	T3	Log-Transformed	T3	
**Adiponectin (pg/ml), N (Cases)**			196 (16)		392 (22)	
**Weighted N (Cases)**			2722 (203)		5224 (181)	
Crude	0.23	1.23 (0.86-1.77)	1.74 (0.85-3.57)	1.29 (0.88-1.87)	1.44 (0.66-3.18)	
Model 1	0.23	0.77 (0.41-1.44)	0.45 (0.13-1.55)	0.91 (0.46-1.80)	0.71 (0.19-2.71)	
Model 2	0.02	0.91 (0.41-2.02)	0.48 (0.11-2.03)	1.08 (0.33-3.58)	0.32 (0.02-4.83)	
**Resistin (pg/ml), N (Cases)**			290 (20)		298 (14)	
**Weighted N (Cases)**			3532 (253)		3614 (168)	
Crude	0.14	**1.97 (1.10-3.53)**	1.64 (0.80-3.37)	1.09 (0.40-2.97)	1.78 (0.75-4.21)	
Model 1	<0.01	**4.53 (1.67-12.32)**	**9.59 (1.45-63.28)**	1.25 (0.37-4.22)	0.91 (0.23-3.71)	
Model 2	0.05	**6.68(2.10-21.21)**	**8.91 (2.72-29.19)**	4.91(0.79-30.34)	1.92 (0.29-12.59)	
**Leptin (pg/ml), N (Cases)**			367 (9)		221 (8)	
**Weighted N (Cases)**			5197 (184)		3381 (126)	
Crude	0.59	**0.61 (0.48-0.79)**	**0.23 (0.10-0.53)**	0.84 (0.65-1.09)	0.55 (0.24-1.27)	
Model 1	<0.01	**0.26 (0.13-0.52)**	**0.02 (0.00-0.23)**	0.86 (0.41-1.78)	1.35 (0.28-6.55)	
Model 2	0.02	0.67 (0.22-2.08)	0.75 (0.03-19.87)	0.38 (0.08-1.90)	1.27 (0.10-15.59)	
**Lp(a) (mg/dl), N (Cases)**			369 (20)		219 (7)	
**Weighted N (Cases)**			4635 (239)		3263 (143)	
Crude	0.09	0.80 (0.57-1.13)	0.66 (0.31-1.38)	0.82 (0.61-1.09)	0.56 (0.23-1.34)	
Model 1	0.43	1.02 (0.47-2.21)	1.32 (0.28-6.12)	1.19 (0.80-1.76)	1.04 (0.22-4.95)	
Model 2	0.13	1.25 (0.53-2.93)	1.37 (0.23-8.07)	1.58 (0.94-2.67)	3.43 (0.34-34.97)	

Model 1: Adjusted for age, gender, education, income, and site.

Model 2: Additionally adjusted for exercise activity, BMI, smoking status, alcohol use, and comorbidity score

T3: 3^rd^ tertile vs. 1^st^ tertile (referent) association.

Bold indicates significance at 0.05 alpha level.

^1^p-value for interaction between log-transformed biomarker and race.

**Table 5:** Hazard ratios (HR) and 95% confidence intervals (CI) for the association between metabolic markers and obesity-related cancer deaths among all participants

	Log-Transformed	T1	T2	T3
**Adiponectin (pg/ml), N (Cases)**		588 (12)	588 (7)	588 (13)
**Weighted N (Cases)**		8386 (192)	8497 (82)	7946 (116)
Crude	1.00 (0.63-1.58)	Referent	0.54 (0.21-1.38)	1.13(0.51-2.50)
Model 1	1.07 (0.66-1.73)	-	0.61 (0.23-1.65)	1.29 (0.52-3.20)
Model 2	1.24 (0.78-1.97)	-	0.92 (0.34-2.51)	2.00 (0.79-5.09)
Model 3	1.04 (0.62-1.74)	-	0.79 (0.29-2.13)	1.47 (0.53-4.06)
**Resistin (pg/ml), N (Cases)**		588 (10)	588 (11)	588 (11)
**Weighted N (Cases)**		8434 (123)	9250 (169)	7145 (99)
Crude	1.27 (0.55-2.92)	Referent	1.00 (0.42-2.39)	1.33 (0.56-3.19)
Model 1	1.21 (0.52-2.82)	-	1.07 (0.44-2.62)	1.23 (0.48-3.15)
Model 2	1.23 (0.59-2.57)	-	1.21 (0.48-3.03)	1.22 (0.48-3.09)
Model 3	1.78 (0.77-4.12)	-	1.42 (0.49-4.15)	1.83 (0.68-4.96)
**Leptin (pg/ml), N (Cases)**		588 (21)	588 (7)	588 (4)
**Weighted N (Cases)**		7750 (219)	8501 (114)	8578 (57)
Crude	**0.59 (0.47-0.75)**	Referent	**0.32 (0.13-0.75)**	**0.19 (0.06-0.56)**
Model 1	**0.60 (0.44-0.82)**	-	**0.31 (0.13-0.77)**	**0.22 (0.06-0.81)**
Model 2	**0.55 (0.39-0.79)**	-	**0.32 (0.13-0.83)**	**0.16 (0.04-0.70)**
Model 3	0.68 (0.44-1.05)	-	0.51 (0.19-1.35)	0.37 (0.07-2.01)
**Lp(a) (mg/dl), N (Cases)**		576 (12)	600 (7)	588 (13)
**Weighted N (Cases)**		8404 (198)	8527 (65)	7899 (127)
Crude	1.06 (0.75-1.50)	Referent	0.57 (0.22-1.44)	1.16 (0.52-2.60)
Model 1	1.12 (0.73-1.70)	-	0.53 (0.20-1.39)	1.38 (0.56-3.38)
Model 2	1.06 (0.65-1.73)	-	0.35 (0.12-1.08)	1.17 (0.47-2.91)
Model 3	1.21 (0.72-2.03)	-	0.46 (0.14-1.50)	1.50 (0.58-3.88)

Model 1: Adjusted for age, gender, education, and income.

Model 2: Additionally adjusted for race.

Model 3: Additionally adjusted for exercise activity, BMI, smoking status, alcohol use, and comorbidity score.

T1: 1^st^ tertile; T2: 2^nd^ tertile; T3: 3^rd^ tertile

Bold indicates significance at 0.05 alpha level.

## DISCUSSION

In a prospective cohort study of Black and White participants, higher levels of pre-diagnostic leptin was independently associated with a lower risk of overall cancer mortality, and with reduced risk of obesity-related cancer mortality. Pre-diagnostic resistin levels were associated with a 6-fold increased risk of cancer mortality but only among Blacks. These results suggest that plasma leptin and resistin may be independent risk markers for cancer mortality, and racial differences may exist in the association between these biomarkers and cancer mortality. Further study is required owing to the relatively small number of cancer mortality cases in this study.

Several epidemiologic studies have examined the role of metabolism-related biomarkers such as adiponectin and leptin in relation to cancer mortality outcomes with conflicting results [2, 4, 12–20]. A recent meta-analysis of 10 studies examining adiponectin and cancer prognosis observed no association with disease free survival, but increased risk of overall mortality [4]. In contrast, another meta-analysis of 9 studies demonstrated reduced risk for colorectal cancer with higher plasma adiponectin levels compared with healthy participants [21], while another meta-analysis reported reduced risk for colorectal cancer with higher adiponectin levels among men only [22]. These conflicting meta-analyses results may be due to heterogeneity between studies in terms of study design, timing of biomarker measurement and sample size limitations. Furthermore, none of these studies directly examined racial differences in the association between biomarkers and cancer mortality. To our knowledge, this is the first study to directly examine racial differences in this topic using multiple biomarkers that were measured at baseline prior to cancer diagnosis, cancer outcomes that were assessed prospectively, and with adjustment for multiple potential confounders also assessed at baseline.

We observed that higher leptin and resistin levels were associated with reduced risk of cancer mortality, an association that was restricted only to Blacks. Other studies have observed that the gene expression level of resistin is significantly enhanced in breast tumors of Black patients [5], and high resistin expression is associated with reduced survival and aggressive tumor characteristics [23]. The lack of association between these biomarkers and cancer mortality among Whites in this study may also explain the heterogeneous results observed in previous meta-analysis where there may have been low numbers of Black study participants and lack of race stratification. In our study, the racial differences in the distribution of leptin and resistin mirror racial differences in the prevalence of diabetes but not BMI. This may also explain why upon adjusting for BMI and other behavioral risk factors, the association of leptin with cancer mortality was attenuated. The association with resistin, however, remained strong and significant after adjusting for BMI, suggesting that resistin may be an important biomarker of cancer mortality among Blacks above and beyond the influence of obesity and/or insulin resistance. Although we did not observe a statistically significant association between adiponectin and Lp(a) and cancer mortality, this area deserves further study, as we may have been under-powered to detect modest associations due to these biomarkers.

The biological mechanisms linking the studied metabolism-related biomarkers and cancer mortality have been the subject of much research. For instance, adiponectin may have anticarcinogenic effects via the activation of AMPK pathways, downregulation of mTOR and antiproliferative and apoptotic effects [1]. However, other studies reported procarcinogenic effects of adiponectin via stimulation of proinflammatory cytokines, inhibition of apoptosis, and dose dependent promotion of colonic cell proliferation [1, 24]. Leptin is a hormone produced by adipose cells that helps to regulate energy balance by inhibiting hunger, opposite to ghrelin, also known as the “hunger hormone” [25]. Leptin reduces food intake by inhibiting neuropeptide Y (NPY) and Agouti-related peptide neurons (AgRP), neurons that are both associated with increase in appetite and decrease in metabolism [25]. Thus, it is plausible that participants with higher leptin experienced a reduced risk of cancer mortality due to lower risk of obesity or less active adipose tissue for a given body-mass index (since we adjusted for this in our analysis). Leptin is also believed to influence cancer prognosis by promoting cancer cell migration and invasion through leptin mediated activation of JAK/STAT3, and by promoting focal adhesion formation, maintenance of stemness and mesenchymal phenotypes in ovarian cancer cells [15]. Resistin is a adipocyte-derived “insulin resistance” hormone originating mainly from macrophages [25]. Higher resistin levels increase glucose uptake in fat cells and increases insulin sensitivity. Resistin has been associated with increased atherosclerotic risk and risk of ischemic stroke [25]. These underlying mechanisms of resistin further explain that increased risk of cancer mortality seen among Blacks with higher baseline levels of resistin are partly due to the prevalence of comorbidities such as obesity and diabetes, however the observed association was statistically independent of these factors. Extensive research has been conducted to elucidate the downstream pathways leading from each specific biomarker to tumor growth and metastasis, and advances in genomics and molecular pathology will continue to generate candidate biomarkers with potential clinical value [26]. The role of metabolic dysregulation, including obesity and insulin resistance, in influencing the levels of these biomarkers, which in turn influences cancer outcomes, may represent the missing link in epidemiologic research. Prior studies have lacked detailed information needed to fully understand Black-White differences in the population distribution of these biomarkers, and race-specific associations with cancer prognosis remains unclear, despite well-characterized racial differences in cancer incidence and mortality.

There were several strengths and limitations applicable to this analysis. First, the use of multiple biomarkers associated with metabolic dysregulation enabled us to examine several risk factors for cancer mortality. Biomarkers were measured at baseline prior to cancer diagnosis, and cancer mortality was assessed during follow-up, enhancing our ability to identify causal factors. While we cannot exclude the possibility that some cancers were present at baseline and this could have altered biomarker levels, participation in REGARDS required that patient were not under active treatment for cancer, so any impact of this bias is likely to be small. The bi-racial composition of the REGARDS cohort, which was retained in our study sample, supported the examination of both race-adjusted and race-stratified analyses. Regarding limitations, first, we had less than 10 years of follow-up data available, potentially resulting in over-representation of highly fatal cancers such as lung, pancreatic and ovarian cancers, and under-representation of breast and prostate cancers. Additionally, the limited number of cancer mortality events precluded analysis of cancer-specific mortality, although we were able to separately examine obesity associated cancer mortality. We also did not have data on cancer incidence. Future studies in the REGARDS cohort with longer follow up data and more cancer outcomes, including with validated cases of cancer incidence, will be needed to address these limitations and provide more definitive data on the molecular epidemiologic underlying racial disparities in cancer mortality.

In conclusion, pre-diagnostic resistin and leptin levels are associated with risk of cancer mortality specifically among Blacks. The utility of these biomarkers in precision cancer prevention and therapy deserves further examination.

## MATERIALS AND METHODS

### Study participants

Data for this study were obtained from the REasons for Geographic And Racial Differences in Stroke (REGARDS) cohort study. REGARDS is one of the largest ongoing national longitudinal cohorts of community-dwelling adults in the United States [27]. Designed to identify contributors to racial and geographic differences in stroke mortality, the cohort included 30,239 participants aged ≥ 45 years at baseline; 45% were male, 41% were Black, and 69% were >60 years old. By design REGARDS oversampled Blacks and residents of the stroke belt, a cluster of states with high stroke mortality: North Carolina, South Carolina and Georgia, plus Tennessee, Mississippi, Alabama, Louisiana and Arkansas [27]. Participants who were not under active treatment for cancer at the time of enrollment were recruited nationally between January 2003 and October 2007, and detailed information about demographics, health behaviors, chronic medical conditions, and physical health status was collected through a computer-assisted telephone interview [27]. Three to four weeks after this interview, a brief physical exam was conducted in the home by centrally trained personnel that included height and weight, blood pressure measurements, blood and urine samples (preferably after a 10-12 hour fast), and medications. Within 2 hours of collection, blood samples were centrifuged and serum or plasma separated and shipped overnight on gel ice packs to the central laboratory at the University of Vermont. On arrival, samples were re-centrifuged at 30,000 xG and 4 degrees Celsius, and either analyzed (general chemistries) or stored at −80 degrees Celsius. During follow-up, participants are contacted by telephone every 6-months to identify any medical events or hospitalizations since the prior contact. The Institutional Review Boards of all participating institutions approved the study.

Biomarker measurements were obtained on 3097 participants from a case-cohort study that included 1,127 participants from a stratified cohort random sample, 649 participants who had an incident stroke, 711 participants who had incident coronary heart disease (CHD), and 296 participants who had incident cognitive impairment. The cohort random sample was selected using stratified sampling to ensure sufficient representation of high-risk groups based on age (20% 45–54 years, 20% 55–64 years, 25% 65–74 years, 25% 75–84 years, and 10% ≥ 85 years), race (50% Black, 50% white), and sex (50% men, 50% women) in order to maximize power to detect age and racial differences [28–30]. Study sample size varied depending on which biomarker was being evaluated, and we presented results here for participants with data on all biomarkers, however results were in the same direction and of similar magnitude when utilizing the entire dataset. Overall, there were 1764 participants (representing a total of 24,829 REGARDS participants) with available biomarkers assays for study analysis and among these participants there were 88 cancer-related deaths (an estimated 1,290 cancer deaths using sampling weights) (Figure 1).

### Main exposure variables

The main exposures of interest in this study were metabolism-related biomarkers including adiponectin, leptin, Lp(a), and resistin, all measured at baseline. Adiponectin and resistin were measured using the Human Serum Adipokine Panel A LINCOplex Kit (Linco Research, Inc.; St. Charles, MO). Leptin was measured by the Human Serum Adipokine Panel B LINCOplex Kit (Linco Research, Inc.; St. Charles, MO). For these three adipokines the intra-assay and inter-assay CVS were 1.4-7.9% and <21%, respectively. Lp(a) was measured with the BNII nephelometer utilizing a particle enhanced immunonepholometric assay (N Latex Lipoprotein-a; Seimens Healthcare Diagnostics, Deerfield, IL); intra-assay CVs 1.8–4.1% and inter-assay CVs 2.0–5.3%.

### Cancer mortality outcome

The primary outcome was death due to any cancer. Cancer mortality was adjudicated using data from death certificates, medical records, interviewed proxies, linkages with the Social Security Death Index (SSDI) and the National Death Index (NDI). Date of death was confirmed using death certificates, SSDI, and/or NDI, and cause of death was adjudicated by a committee of experts using all available information as recommended by national guidelines [31, 32]. As a secondary outcome, we examine obesity-related cancer deaths defined as cancers of the breast, colorectal, kidney, pancreas, stomach, endometrial, and esophagus [33]. Follow-up time for each participant was calculated from the enrollment date through date of cancer death, death, or last telephone follow-up through December 31, 2012, when adjudication of death was complete for the cohort.

### Study covariates

Baseline demographic variables included age, race, sex, annual household income, and education. Health behaviors included smoking status, alcohol use, and exercise activity. Medication use included aspirin and statins. Chronic baseline medical conditions considered included atrial fibrillation, chronic lung disease, chronic kidney disease, coronary artery disease, deep vein thrombosis, diabetes, dyslipidemia, hypertension, myocardial infarction, obesity, peripheral artery disease, and stroke. We created an individual level comorbidity score based on the sum of total number comorbidities. Detailed descriptions of characteristics are in Supplementary Tables 1 and 2.

### Statistical analysis

We applied statistical weights to account for the cohort sampling scheme in order to ensure sufficient representation of high-risk groups and reflect the age, sex, and race distribution of 29,653 participants of the REGARDS cohort, and enhance generalizability of study results to the entire cohort [28]. We performed analysis by tertiles and log-transformations of each metabolic biomarker (due to non-normal distributions). We compared baseline characteristics by tertiles of each biomarker, and presented the weighted participant totals, proportions, medians with interquartile ranges (IQRs), and means with standard deviations (for comorbidity score). We used Chi-square and Kruskal-Wallis tests as appropriate. We performed Cox Proportional Hazards models to estimate hazard ratios (HRs) with robust sandwich estimators to calculate the 95% confidence intervals (CIs) for the association between metabolism biomarkers (both log-transformed and tertiles) and cancer mortality, accounting for population weighing. We sequentially adjusted the models for 1) age, sex, education, income, and cancer site, 2) race, 3) exercise activity, BMI, smoking status, alcohol use, and comorbidity score. We repeated the above analyses stratified by race/ethnicity and also by obesity status. Lastly, we examined the association between metabolic biomarkers and risk of obesity-related cancer death. Individuals were censored at the time of death, loss to follow-up, or the end of cancer mortality ascertainment (December 31, 2012). SAS version 9.4 was used for all statistical analysis. We considered two-sided p values <0.05 as statistically significant.

## SUPPLEMENTARY MATERIALS TABLES


