# Supplementary material for: Pre-diagnostic biomarkers of metabolic dysregulation and cancer mortality
Source: Oncotarget. 2018 Feb 23;9(22):16099–109. doi: 10.18632/oncotarget.24559 (PMC5882320; doi:10.18632/oncotarget.24559)
Supplement: Supplementary file 1 [file oncotarget-09-16099-s001.pdf]

Pre-diagnostic biomarkers of metabolic dysregulation and cancer mortality

Supplementary Table 1: Detailed definitions and technical information for sociodemographics, health behaviors, chronic medical conditions, and medication use

Characteristic	Definition and/or Technical Information
**Sociodemographics**	
Age	Age in years
Gender	Male, female
Race	Black, white
Education	Participant reported:<br>- Less than high school<br>- High school graduate<br>- Some college<br>- College or higher<br>- Missing
Income	Participant reported:<br>- <$20k<br>- $20k-$34k<br>- $35k-$74k<br>- ≥$75k<br>- Missing (not reported)
**Health Behaviors**	
Smoking Status	Participant reported:<br>- Current<br>- Past<br>- Never
Alcohol use	Participant reported:<br>- None<br>- Moderate (up to 1 drink per day for women or 2 drinks per day for men)<br>- Heavy (>1 drink per day for women and >2 drinks per day for men) [1].
Exercise activity	Participant reported:<br>- None<br>- 1 to 3 times per week<br>- 4 or more times per week.

Supplementary Table 2: Chronic Medical Conditions

Characteristic	Definition and/or Technical Information
Atrial Fibrillation	Participant reported history of atrial fibrillation.
Chronic Lung Disease	Participant use of pulmonary medications (beta agonists, leukotriene inhibitors, inhaled corticosteroids, combination inhalers, ipratropium, cromolyn, aminophylline and theophylline) as a surrogate for chronic lung disease.
Coronary Artery Disease	Participant reported history of myocardial infarction, coronary artery bypass grafting, or cardiac angioplasty or stenting, or baseline electrocardiographic evidence of myocardial infarction.
Diabetes	Fasting glucose ≥126 mg/L (or a glucose ≥200 mg/L for those not fasting) or participant reported use of insulin or oral hypoglycemic agents.
Deep Vein Thrombosis	Participant reported history of deep vein thrombosis.
Dyslipidemia	Low-density lipoprotein cholesterol >130 mg/dL or participant reported use of lipid lowering medications.
Hypertension	Systolic blood pressure ≥140 mm Hg, diastolic blood pressure ≥90 mm Hg, or participant reported antihypertensive agent use.
Myocardial Infarction	Participant reported history of myocardial infarction or baseline electrocardiographic evidence of myocardial infarction.
Obesity	or {body mass index ≥30 kg/m^2}.
Peripheral Artery Disease	Participant reported history of lower extremity arterial bypass or leg amputation.
Stroke	Participant reported history of stroke or transient ischemic attack.
**Medications**	
Aspirin use	Participant use of aspirin.
Statin use	Participant use of statins.

REFERENCE

1. National Institute on Alcohol Abuse and Alcoholism. Helping Patients Who Drink Too Much, a Clinician's Guide [http://pubs.niaaa.nih.gov/publications/Practitioner/CliniciansGuide2005/guide.pdf].
